# Efficient Plant Growth-Promoting (PGP) Native *Actinomycetes*-Formulated Consortia Mode and Assessed Shelf Life Using Low-Cost Dynamic Media

**DOI:** 10.4014/jmb.2409.09040

**Published:** 2025-02-25

**Authors:** Thirupathi Seenivasa Moorthy, Raju Kannan, Rekha Thiruvengadam, Shine Kadaikunnan, Jamal M. Khaled, Baskar Venkidasamy, Muthu Thiruvengadam

**Affiliations:** 1 Department of Biotechnology, Kalasalingam Academy of Research and Education (KARE), Krishnankoil 626126, Tamil Nadu, India; 2 Department of Agriculture, Kalasalingam Academy of Research and Education (KARE), Krishnankoil 626126, Tamil Nadu, India; 3 Molecular Microbiology Laboratory, Department of Community Medicine, Saveetha Medical College and Hospitals, Saveetha Institute of Medical and Technical Sciences, Saveetha University, Chennai 602105, India; 4 Department of Botany and Microbiology, College of Science, King Saud University, P.O. Box 2455, Riyadh 11451, Saudi Arabia; 5 Centre for Bioscience and Biotechnology, Saveetha Dental College and Hospitals, Saveetha Institute of Medical and Technical Sciences, Saveetha University, Chennai 600077, India; 6 Department of Applied Bioscience, College of Life and Environmental Science, Konkuk University, Seoul 05029, Republic of Korea

**Keywords:** *Actinomycetes*, carrier material, compatibility, consortium, IoT device, shelf-life

## Abstract

Various *Actinomycete* isolates were collected from the microbial germplasm, including *Streptomyces rameus* KAC3 (PP550146), *Streptomyces bangladeshensis* KAC4 (PP177363), *Streptomyces mutabilis* KAC6 (PP177364), *Microbacterium arabinogalactanolyticum* KAC8 (PP177365), *Streptomyces calvus* KAC10 (PP177366), *Streptomyces werraensis* KAC11(PP177366), and *Streptomyces gancidicus* KAC12 (PP177389). Three consortia formulations were then selected based on their compatibility with plant growth-promoting rhizobacteria (PGPR) properties under laboratory conditions: C_1_ consortium (KAC3, KAC10 and KAC11), C_2_ (KAC4, KAC8 and KAC10), and C_3_ (KAC11 and KAC12). In this study, we explored the use of locally available, cost-effective carrier materials, such as wood ash (NPK ratio: 1.72:0.86:3.6), charcoal (NPK ratio: 0.93:23.68:0.8), eggshell powder, a surfactant agent (SDS + CTAB), and vermi-compost (NPK ratio: 3.5:0.71:0.6). The CFU counts of *Actinomycete* isolates in the consortia were calculated over six months at different temperatures (28 and 35±2°C) using these low-cost materials. The C_1_ consortium showed the highest stability in maintaining viable populations, whereas C_2_ and C_3_ showed the best performance in the combined-ingredient carrier material. The efficacy of C_1_ was monitored using an IoT device and further validated under different temperature conditions for six months. The data showed that temperature variation affected *Actinomycete* growth, with an optimal viability at 28±2°C. Moreover, increases in temperature led to higher CO_2_ levels and decreased moisture and humidity, yet the *Actinomycete* population remained stable. This highly efficient C_1_ consortium formulation is a promising eco-friendly bioinoculant for sustainable agriculture.

## Introduction

The Actinobacteria group of gram-positive bacteria plays an important role in heterogeneous soil systems and is involved in biogeochemical cyclic processes [1]. Densities of soil microorganism distribution, especially *Actinomycetes*, are highly diverse, ranging from 10% to 50% under abiotic and biotic conditions, because *Actinomycete* spores actively proliferate under stress conditions and promote soil health improvement [2]. *Streptomyces* is the predominant organism in the lithosphere and produces economically important active compounds (antibiotics, anti-parasitic, antimicrobial, and immunosuppressive). *Actinomycetes* contribute to soil health and promote plant growth through the production of auxins and cytokines via various mechanisms [3, 4].

*Actinomycetes* are unique and significant contributors to plant development and yield increases because, in conjunction with appropriate carrier materials, they may remain in the soil for extended periods and participate in several aspects of plant growth [5]. *Actinomycetes* have recently received considerable interest as plant growth promoters because of their high antibacterial capability and soil-dominant saprophytic nature [6]. To guarantee that the appropriate amount of an *Actinomycete* population remains alive for the inoculants to be sustained effectively, it is crucial to ascertain the length of bacterial survival in the corresponding carrier materials. In addition, the chosen carrier materials should be reasonably priced, capable of withstanding adverse environmental conditions, and easily dissolve in water to allow the release of *Actinomycetes* [7].

Soil carrier materials (peat, coal, and clay) can be classified as plant waste products (compost, soybean meal, and farmyard manure), and inert materials (talcum powder, vermiculite, perlite, alginate, and other inorganic minerals) [4]. Thus, formulations that include one or more beneficial bacterial strains (or species), combined with an affordable and convenient carrier material, have been studied to efficiently distribute bioinoculants to desired crops [8]. Various carrier materials, such as talc, peat, and karnalite, although used in agriculture, are expensive, scarce, and environmentally harmful. Bagasse, sawdust, charcoal, and wood ash are favored microbial inoculants because of their large surface area, moisture retention, affordability, and eco-friendliness. Their capacity to sustain high plant growth-promoting rhizobacterial (PGPR) densities is crucial for effective plant development. The quantity of injected microorganisms significantly affects the effectiveness of microbial inoculation in promoting plant growth. Therefore, ensuring adequate bacterial survival in carriers is vital [4]. Bagasse, sawdust, charcoal, and wood ash, which are affordable and water-soluble, can endure diverse environmental conditions and facilitate bacterial release for sustained inoculation efficacy [9]. Adhesive materials, such as adjuvants like sodium dodecyl sulfate (SDS) and cetyl trimethyl ammonium bromide (CTAB), maintain the bond between microbes and carriers. Charcoal, vermicompost, eggshell powder, and wood ash are preferred owing to their adhesive properties. In addition, the quantity of injected microorganisms significantly influences their effectiveness, necessitating the assessment of bacterial survival in carriers [10].

An *Azotobacter chroococcum*-based biofertilizer formulation was optimized using different carrier materials by sterilization using autoclave and gamma irradiation for peat moss, a mixture of peat moss and vermiculite 1:2 (w/w), wheat bran, rice husk, clay, and sodium alginate. The results showed locally available low-cost materials to be suitable as carriers capable of adapting bacterial species for a long shelf life [11, 12]. Similarly, rhizobium inoculants were evaluated in a granular formulation using peat as a carrier material for compatibility with physical, chemical, and biological parameters [13]. Commercially produced rhizosphere-efficient strains using cark industry waste residues as a carrier material have been used for the successful production of inoculants without environmental problems and long shelf life [14].

While there are problems with the shelf life of plant growth-promoting organisms in the long term, plant growth-promoting rhizobacteria (PGPR) can enhance plant growth and protect against stresses, maintaining their viability and effectiveness as inoculants over extended storage periods. In the agricultural sector, *Actinomycetes* are the dominant habitat species of *Streptomyces* commercially produced for plant growth promotion and are merely free-living soil organisms recognized for their complex interactions with plants and other organisms [2, 15]. Ingredient optimization can be accomplished using charcoal, vermicompost, eggshell powder, SDS and CTAB and wood ash, which have been shown to work best for plant growth *Actinomycete* inoculants. They can sustain a high density of PGPR and retain their viability because of their wide surface area, excellent moisture-holding capacity, affordability, and environmental friendliness. Therefore, our goal in this study was to compare the sustainability, viability, and suitability of five different carriers for the survival of PGPR isolates at room temperature. Additionally, external parameters such as temperature, moisture, humidity, and CO_2_ level were monitored on the Internet of Things (IoT) to investigate the possibility of long-term shelf life of the isolates in various carrier materials during storage.

## Materials and Methods

### Survey and Addressing of Carrier Materials

**Fig. 1:**
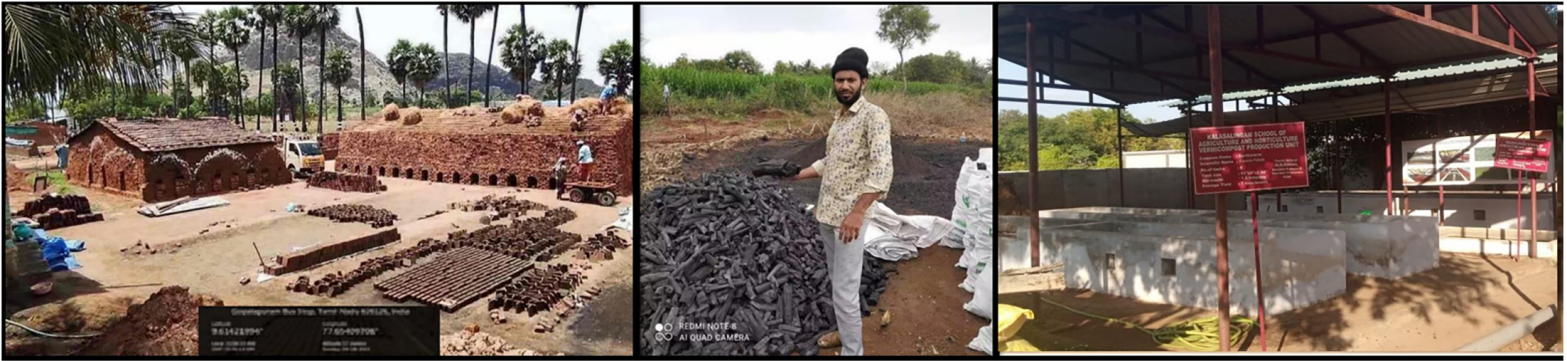
GPS location photography of carrier material available in Virudhunagar.

**Fig. 2:**
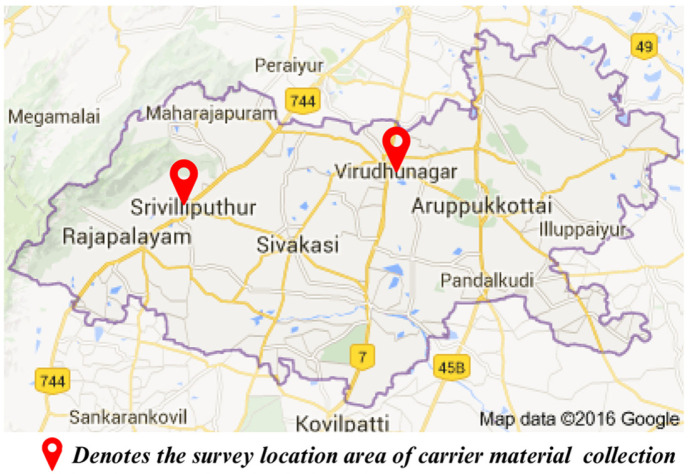
Overall location of carrier material availability in Virudhunagar.

Locally available industrial and domestic waste materials were selected (Figs. 1 and 2) for *Actinomycete* inoculant carrier materials, including wood ash (450 bricks-industry units), charcoal (*Prosopis juliflora* occupies 40% of the geographical area in Virudhunagar district), eggshell powder (hostels, restaurants), vermi-cost (Kalasalingam University), and adhesive (resistant to microbial degradation). Carrier material-rich macro (N, P, K, Ca, Mg) and micro (Zn, Cu, Mn, Fe) nutrient natural element resources and slow nutrient release capability of each carrier material with high affinity properties of adhesive will enhance inoculant viability for a long duration and simultaneously induce soil fertility with water-holding capacity (Table 1).

### Collection of PGP *Actinomycetes* Germplasm

Plant growth-promoting *Actinomycetes* were collected from the Agricultural Microbiology Laboratory, Department of Agriculture, Kalasalingam Academy of Research and Education, Krishnankoil, Virudhunagar District, Tamil Nadu, India (Table 2).

### Physical, Chemical and Microbial Analysis of Carrier Materials

The physical and chemical properties of the collected carrier materials include the pH and electrical conductivity (EC). The pH of the carrier material extract was determined potentiometrically using an ORION ion analyzer with a pH and conductivity electrode [21].

Organic carbon was estimated using a rapid titration method [22] involving oxidizing the organic matter in carrier materials using a chemical oxidant, typically potassium dichromate (K_2_Cr_2_O_7_), in the presence of sulfuric acid (H_2_SO_4_). The reaction releases carbon dioxide (CO_2_), which is indirectly measured by titrating the remaining dichromate with a reducing agent, such as ferrous ammonium sulfate. The amount of titrant used reflects the amount of organic carbon present in the carrier material and offers a fast and reliable estimate. Available phosphorus was determined using the Olsen method [23] for carrier materials, particularly under neutral-to-alkaline conditions. In this method, a sodium bicarbonate (NaHCO_3_) solution (0.5 M) was used as the extractant and the soil sample was shaken with the solution at a pH of 8.5. After the extraction, the phosphorus concentration in the filtrate was determined calorimetrically using the molybdate blue method. Available potassium was determined by the ammonium acetate extractable method using a flame photometer [23] to extract potassium (K) from the carrier material using 1 N ammonium acetate (NH_4_CH_3_CO_2_) at a pH of 7.0. The carrier material was mixed with an ammonium acetate solution, shaken, and filtered. The filtrate, which contained the extracted potassium, was analyzed using a flame photometer. In a flame photometer, potassium ions emit light at a wavelength of 766.5 nm in the visible spectrum. These ions are excited in a flame, which enables the quantification of potassium levels in the soil. Typically, 25 ml of 1 N ammonium acetate is used per 5 g of carrier material for extraction, and available nitrogen by the alkaline permanganate method [24], involves treating the carrier material with an alkaline potassium permanganate solution (KMnO_4_) under controlled conditions. The solution oxidizes organic matter, releasing ammonium (NH_4_^+^), which is then measured as a proxy for available nitrogen. Typically, 0.32% KMnO_4_ solution is used, along with sodium hydroxide (NaOH) to create the alkaline medium. The carrier material was heated with the solution, and the evolved ammonia was collected and titrated to determine nitrogen levels.

### In Vitro Assessment of *Actinomycete* Isolates for PGP Traits

Isolated *Actinomycetes*, including, *Streptomyces rameus* (KAC3), *Streptomyces bangladeshensis* (KAC4), *Streptomyces mutabilis* (KAC6), *Microbacterium arabinogalactanolyticum* (KAC8), *Streptomyces calvus* (KAC10), *Streptomyces werraensis* (KAC11), and *Streptomyces gancidicus* (KAC12) were investigated for their PGP properties, such as the production of siderophores, indole acetic acid (IAA), ammonia, mineral solubilization (calcium phosphate), and potassium under *in vitro* conditions.

### Solubilization of Phosphate

Phosphate solubilization by the isolates was evaluated based on their ability to solubilize inorganic phosphates. Pikovskaya’s agar medium, containing calcium phosphate as the inorganic form of phosphate, was used in the assay. A loopful of *Actinomycete* isolates were streaked onto plates and incubated at 28°C for 4-5 days. The appearance of a transparent halo around the *Actinomycete* colony indicated the phosphate-solubilizing activity of *Actinomycetes*, followed by previously described [25].

### Solubilization of Potassium

The ability of the isolates to solubilize potassium was tested by spot inoculation of *Actinomycete* isolates on Aleksandrov medium, as per the method described in previous study [25], and plates were incubated at 28±2°C for 3–5 days. The formation of a clearance zone around the spots indicates potassium solubilization.

### Colorimetric Analysis of IAA Production

Indole acetic acid (IAA) production was detected as previously described [26]. *Actinomycete* isolates were grown for 72 h in Actinomyces broth at 30°C. Fully grown cultures were centrifuged at 3,000 ×*g* for 30 min. The supernatant (2 ml) was mixed with 2 drops of orthophosphoric acid and 4 ml of Salkowski reagent (50 ml, 35% perchloric acid, 1 ml 0.5 M Ferric chloride (FeCl3) solution). The development of a pink color indicated IAA production.

### Siderophore Production

Siderophore production was assayed qualitatively using Chrome Azurol S (CAS) blue agar [21]. CAS agar plates were spot inoculated with each *Actinomycete* isolate, and the development of an orange halo zone around the colonies was recorded as the measurement of siderophore production.

### Testing Compatibility between *Actinomycete* Isolates

Collected *Actinomycete* isolates were subjected to an *in vitro* compatibility assay using the cross-streak method, where each isolate was streaked perpendicularly to other isolates on *Actinomycete* isolation agar (AIA) medium. All plates, including the control, were incubated at 28°C for 4 days. After incubation, positive results were observed for inter-junction in the merger of *Actinomycete* growth for compatibility with each other in the plate [27].

### Consortium Preparation in Dynamic Media

Based on the compatibility test, the active consortia were prepared and designated as C_1_, C_2_, and C_3_ in Table 7. Initially, a single colony from each *Actinomycete* isolate was inoculated into Actinomyces broth and incubated at the optimum pH and temperature overnight with continuous shaking (120 rpm). Growth measurements were adjusted based on optical density (OD) at 600 nm. Furthermore, consortia formulations adapted in our laboratory for equivalent amounts of 1,000 μl (10^3^) of active culture from each isolate were transferred to 100 ml of sterile autoclaved formulation, that is, a low-cost and easily available, nutritional source for bacterial growth (vermi-wash, 50 ml; cow dung slurry, 25 ml; jaggery, 15 ml; black jaggery, 10 ml) and mixed to prepare the individual consortium and incubated at 30°C for 5 days with continuous shaking (120 rpm), followed by previously described [28].[Table T3]

### Solid-Based Carrier Bioformulation with *Actinomycete* Isolates

Liquid cultures of the *Actinomycete* consortium (100 μl) and individual isolates were transferred to 300 ml Actinomyces broth to scale up and constitute an active consortium; 300 ml of the prepared active consortium was centrifuged at 10,000 ×*g* for 10 min. Then, *Actinomycete* pellets were incorporated with 10 g of 100 g sterilized combined ratio of carrier material (vermi-cost: 50 g; charcoal: 20 g; wood ash: 10 g; eggshell powder: 20 g; adhesive material SDS and CTAB mix in a ratio of 1:1) under aseptic conditions. The mixture was vortexed for 45 min to homogenously mix the carrier material, and the *Actinomycete* cells were dried at room temperature (28±2°C). After complete drying, the individual and combined-based formulations were packed in clean airtight sterilized packets and sealed separately. The experiments were performed in triplicate. The prepared bioformulations were stored at room temperature 28±2°C and 35±2°C [28].

### Shelf Life Assessment of Solid-Based Carrier Material

The viability of *Actinomycete* cultures in the formulation was confirmed by the serial dilution plating method. The formulated product was validated at two different temperatures for incubation under controlled conditions for up to 6-month periods to assess the viability and population status of the *Actinomycete* isolates. For this purpose, 10 g of packed C_1_, C_2_, and C_3_ consortium formulations were prepared, and a 100 ml stock suspension was prepared in a 250 ml conical flask under aseptic conditions. Serial dilutions of the stock suspension were transferred to test tubes up to 10^-6^. The diluted suspensions were transferred to an *Actinomycete* isolation agar medium using the spread plate method, and the plates were incubated at 28±2°C and 35±2°C. Viability was evaluated initially on the 2^nd^ and 4^th^ day. The final CFU/g was calculated as the average of three results obtained from triplicate plate counts [28]. After a predetermined time, the smallest colonies were obtained. As a result, we are able to determine the shelf life or period in which it is best used. Thus, this technique simplifies the determination of the shelf life of a specific biofertilizer sample and provides precise information regarding its application [15].

### Digitalized Storage with IoT-Based Monitoring of Physical Parameters

**Fig. 3:**
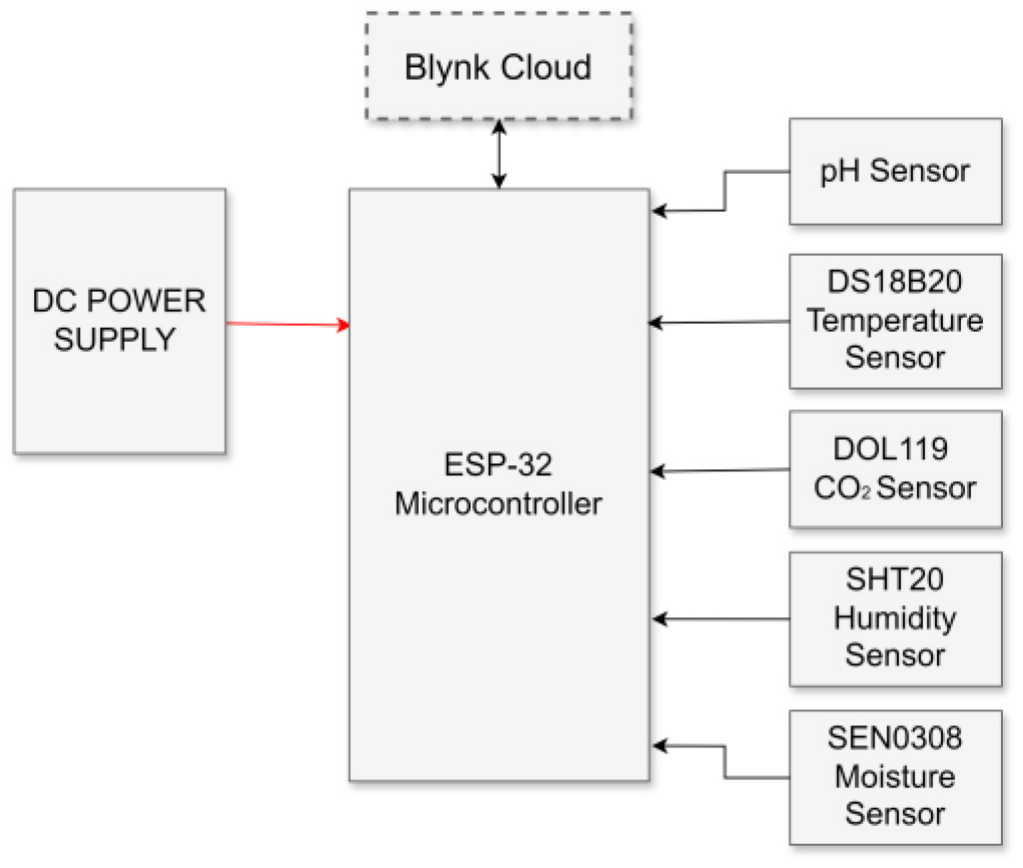
Block diagram of the IoT device.

The physical parameters of the stored formulated carrier material with the inoculant, as referenced in [29], were monitored using an IoT device. Fig. 3 shows the block diagram of a smart environmental monitoring system using an ESP-32 microcontroller. The system integrates various sensors to monitor environmental parameters and communicate data to the Blynk Cloud for remote monitoring. Each component of a diagram plays a critical role in ensuring that the system functions effectively. The DC power supply provided the necessary electrical power to the ESP-32 microcontroller and all the connected sensors. The ESP-32 microcontroller is the core of the system and is responsible for processing the data collected by the sensors and managing communication with the Blynk Cloud. The Blynk Cloud is a platform that allows the remote monitoring and control of IoT devices. In this system, ESP-32 sends sensor data to the Blynk Cloud via a Wi-Fi connection. Blynk Cloud provides a user-friendly interface accessible from smartphones, tablets, or computers, where users can monitor real-time data, s*et al*erts, and control devices remotely. The bidirectional arrow between ESP-32 and Blynk Cloud in the diagram indicates that ESP-32 can send data to and receive commands from the cloud platform, enabling a fully interactive system. Table 4 provides a detailed explanation of each block, its function, and the significance of directional arrows.

### Statistical Analysis

For the experiments, IBM SPSS statistics was used as the statistical software for analysis of variance (ANOVA, version 29.0.2.0), and means were separated using Duncan's multiple range test (*p* = 0.05). All experiments were performed in triplicate, and the data are presented as mean values±standard deviation (SD). The correlation coefficients between *Actinomycete* isolate counts at the two different temperatures were calculated (*p* = 0.05). The results were considered statistically significant at the 95% confidence interval (*p* = 0.05).

## Results

### Selection and Characteristics of Carrier Materials

Table 5 presents the characteristics of the different carrier materials and reveals the notable differences in their properties. In that analysis of pH, electrical conductivity (EC), available Nitrogen, Phosphorus and Potassium (NPK) ratio, total carbon content, and carbon-to-nitrogen (C:N) ratios were demonstrated for agricultural applications.

Wood ash has high carbon content, a balanced carbon-to-nitrogen ratio, a reasonable amount of nitrogen, phosphorus, and potassium, and is strongly alkaline and salinized. Charcoal has very high salinity and a somewhat acidic pH. It also has very high phosphorus content and low quantities of potassium and nitrogen. Its high carbon-to-nitrogen ratio suggests that it contains a significant amount of carbon compared with nitrogen. Because it is neutral, eggshell powder has low salinity and a high nutrient content. The vermi-cost method has a moderate salinity level, an almost neutral pH, and a relatively low carbon-to-nitrogen ratio. It also offers a fair amount of nitrogen in relation to its potassium and phosphorus content. The surfactants SDS and CTAB, both mildly acidic, lack nutrients, and are used as adhesives. According to the methodology, the solid-based carrier bioformulation process for a unique formulation is very high in the alkaline range but can be adjusted to neutral (pH 6.5 – 7) conditions using lime (CaO).

### In Vitro Assessment of PGP Traits for *Actinomycete* Isolates

**Fig. 4:**
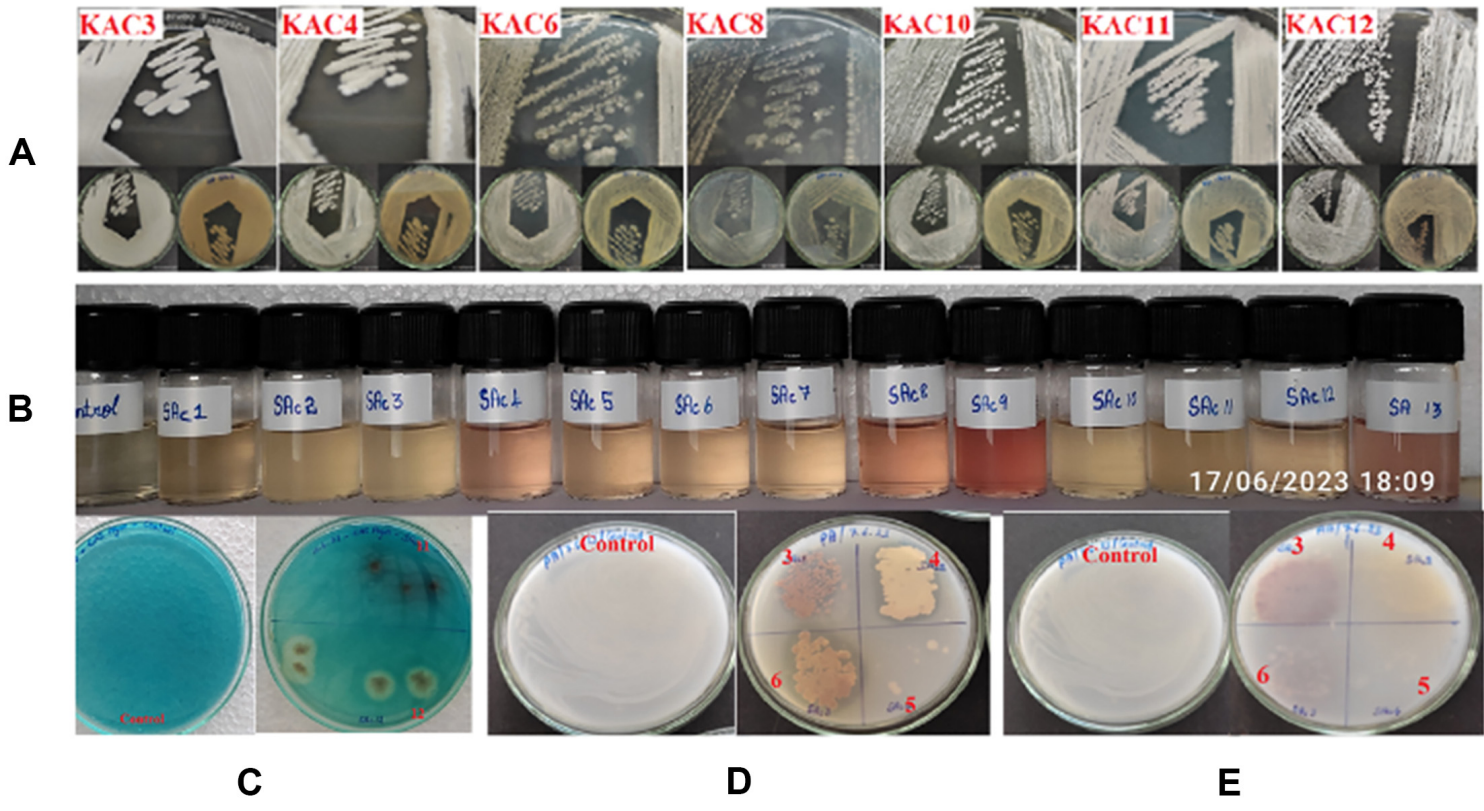
In vitro qualitative assessment of PGP traits. All experiments were performed in triplicate and repeated thrice under the same conditions. (**A**) Aerial and substrate growth in AIA medium. (**B**) IAA production, (**C**) siderophore production, (**D**) phosphate solubilization, and (**E**) potassium solubilization.

Seven isolates were further screened for PGPR traits (Table 6 represents the *in vitro* assessment of PGP traits), including IAA, siderophore, phosphate, and potassium solubilization. Among them, seven isolates (KAC3, KAC10, KAC11, and KAC12) were positive for IAA, siderophore, phosphate, and potassium solubilization. Among the seven isolates, KAC3, KAC10, and KAC11 showed extensive zone formation for siderophore, phosphate, and potassium solubilization. Among the seven isolates, KAC3 and KAC11 produced IAA with dark-pink color changes in fully grown cultures under laboratory conditions. Isolate KAC12 showed a moderately positive response for the PGP traits. Among the seven isolates screened, three isolates, KAC3, KAC10, and KAC11, exhibited positive responses to all the *in vitro* PGPR characteristics studied (Fig. 4).

### Compatibility Studies and Consortium Selection

**Fig. 5:**
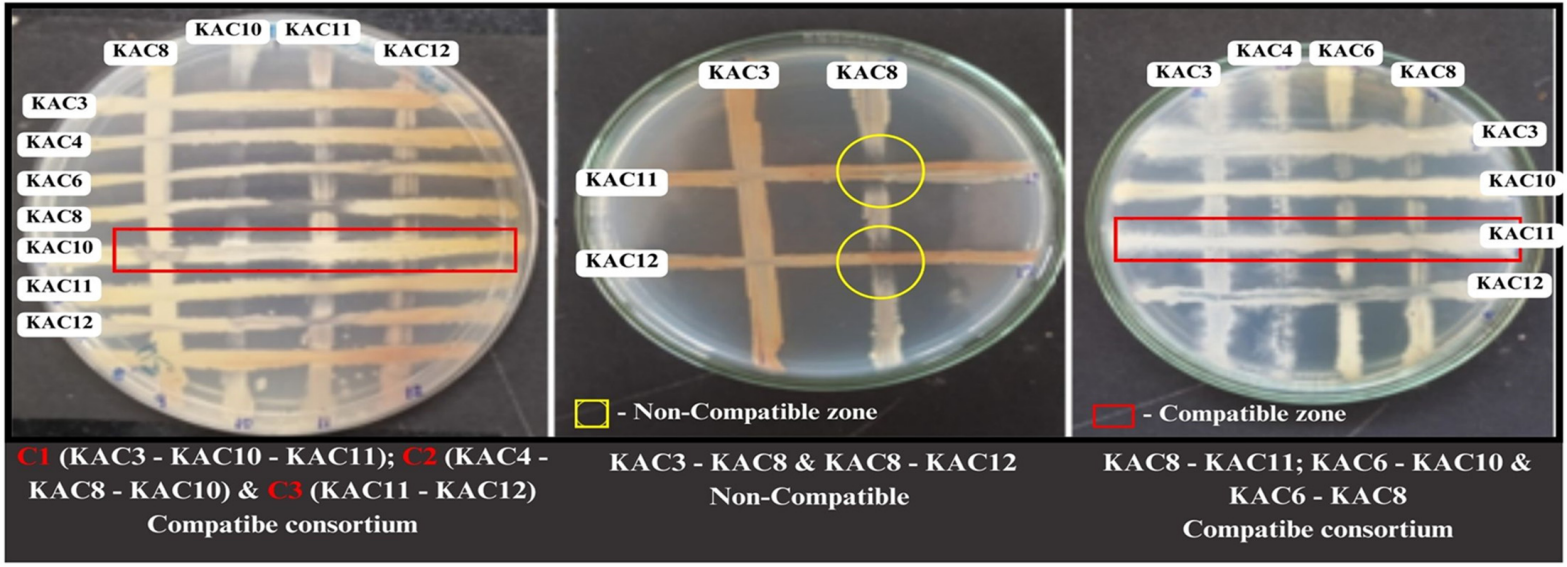
Compatibility assessments of *Actinomycete* isolates. All experiments were performed in triplicate and repeated thrice under the same conditions.

Seven *Actinomycete* species isolated from the soil environment in the Virudhunagar District, Tamil Nadu, were collected. The seven *Actinomycete* isolates were then used for compatibility assays under laboratory conditions: KAC3 (PP550146), KAC4 (PP177363), KAC6 (PP177364), KAC8 (PP177365), KAC10 (PP177366), KAC11 (PP177374), and KAC12 (PP177389) (Fig. 5). The results observed for the two isolates were compatible (KAC8 -KAC11, KAC6 - KAC10 and KAC6 - KAC8) and three isolates were positive for compatibility (KAC3 - KAC10 -KAC11 and KAC4 - KAC8 - KAC10). Three isolates mutually interacted and were selected for further formulation and labeled C_1_ (KAC3 - KAC10 - KAC11), C_2_ (KAC4 - KAC8 - KAC10), and C_3_ (KAC11 - KAC12) consortia (Table 7).

### Shelf Life of Individual *Actinomycete* Isolates in Combined Carrier Materials

In our study, the survival of seven different *Actinomycete* isolates (KAC3, KAC4, KAC6, KAC8, KAC10, KAC11, and KAC12) in a combined carrier material over six months at 28±2°C and 35±2°C, was measured in colony-forming units per gram (CFU/g).

In observation of viability at 28±2°C at the start (Month 0), all isolates exhibited relatively high CFU counts, ranging from 10.07 to 10.23 log CFU/g. However, over time, a gradual decline in CFU counts was observed across all isolates. KAC3 maintained the highest CFU count throughout the six-month period, starting at 10.17 log CFU/g and declining to 8.53 log CFU/g by Month 6, showing a more gradual reduction compared to other isolates. Meanwhile, KAC10 showed a moderate decline from 10.10 log CFU/g at the start to 8.04 log CFU/g by the end of the observation period. KAC11 displayed a similar pattern, decreasing from 10.20 to 8.01 log CFU/g. When comparing these isolates, KAC3, KAC10, and KAC11 showed the highest resilience, whereas KAC4, KAC6, KAC8, and KAC12, which experienced moderate levels of viability, are shown in Table 8.

Upon observation of viability at 35±2°C, at Month 0, all isolates had high CFU counts, ranging from 9.83 to 10.13 log CFU/g. KAC3, starting at 10 log CFU/g, showed a relatively moderate decline to 7.83 log CFU/g by Month 6. KAC10, which started at 10.13 log CFU/g, showed a reduction to 7.64 log CFU/g by Month 6. The KAC11 cells followed a similar trajectory, starting at 10 log CFU/g and decreasing to 7.53 log CFU/g. All isolates experienced a decline in CFU counts over time, with KAC6 showing the most dramatic decrease in viability, and KAC3, KAC10, and KAC11 demonstrating better survival at the higher temperature of 35±2°C. KAC8 and KAC12 showed moderate decline, with KAC4 experiencing a relatively steep decrease. The data suggest that temperature plays a significant role in the survival of these isolates, with faster viability loss observed at 35±2°C compared to lower temperatures, as shown in Table 9.

### Shelf Life of Consortia *Actinomycete* Isolates in Combined Carrier Materials during Storage Period

The populations of the *Actinomycete* consortia (consortia 1, 2, and 3) in the combined bioformulation stored at two different temperatures (28±2°C and 35±2°C) for up to six months, measured in colony-forming units per gram (CFU/g), are shown in Table 10. At 28±2°C, C_1_ initially had the highest CFU count (10.27 log CFU/g), which decreased steadily over the six-month period to 8.48 log CFU/g. This consortium demonstrated relatively better stability than the others at this temperature. C_2_ started at 10.03 log CFU/g and saw a significant decline to 7.22 log CFU/g by Month 6, indicating the sharpest drop in population among the three consortia. C_3_, beginning at 10.13 log CFU/g, declined to 7.78 log CFU/g by the end of the six months, showing a moderate loss in viability. At 35±2°C, all consortia showed a pronounced reduction in the CFU counts. C_1_, starting at 10.07 log CFU/g, had a slower decline compared to the other two consortia, finishing at 7.65 log CFU/g by month 6. C_2_, which began at 9.83 log CFU/g, exhibited the sharpest drop in population, declining to 6.47 log CFU/g after six months, indicating significant temperature sensitivity. C_3_ followed a similar pattern with an initial count of 10.1 log CFU/g, decreasing to 7.02 log CFU/g by month 6.

### IoT-Based Monitoring of External Parameters for Stored Bioinoculant-Assessed Carrier Materials

**Fig. 6:**
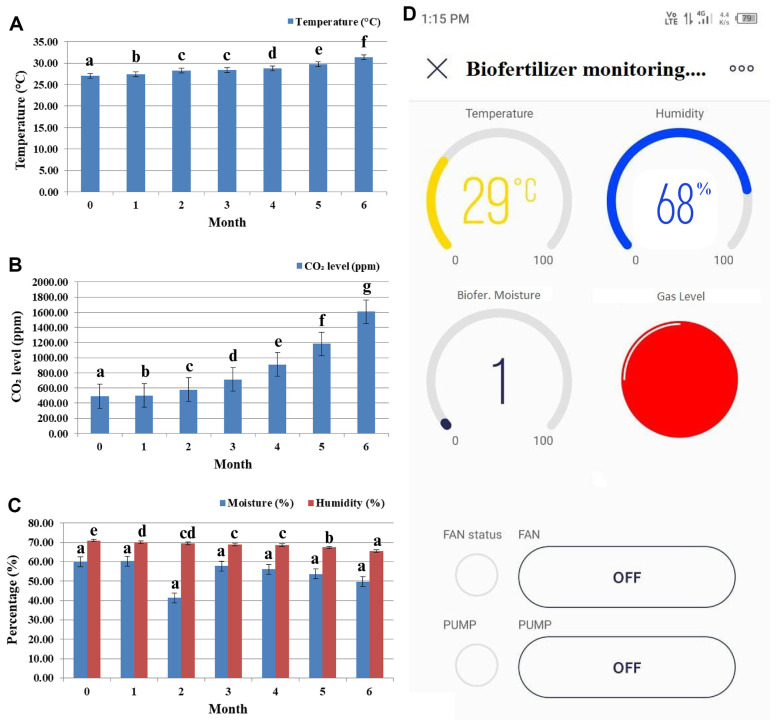
IoT-based monitoring of (A) temperature (°C), (B) CO_2_ levels (ppm), (C) moisture and humidity (%), and (D) design of monitoring applications in stored bioinoculant-assessed carrier materials.

Four widgets from the Blynk program were employed in this study: a gauge for moisture and humidity content, labeled value for temperature, display value for pH level, and notification system for the color sensor. Software testing was conducted concurrently with testing of each sensor connected to the Blynk application. Monitoring the external parameters using the sensor-applied IoT system predicts the accuracy of the temperature increase from the 1^st^ month to the end of the 6^th^ month (27 to 31°C), with a gradual increase in the CO_2_ level (491.48 − 1,606.33 ppm), moisture level reduction (60 to 49.67%), and humidity (71 to 65.49%). The external parameters were monitored through IoT using Blynk application under *in vitro* conditions, as shown in Fig. 6.

## Discussion

The results of three types of carrier materials used (peat, rice husks, and local kaolin) showed after 28°C storage for eight weeks that the viable rhizobia count was high in peat compared to that of rice husk and kaolin [35]. Investigation of vermin-cast combined with lignite as a carrier substrate for biofertilizers showed viable cell counts exceeding 10×10^6^ CFU/g for *Azotobacter chroococcum*, *Bacillus megaterium*, and *Rhizobium leguminosarum* after ten months [18]. Lignite alone showed no viable cells after six months. Charcoal combined with glycerol exhibited significantly higher microbial counts over a three-month storage period, indicating enhanced stability and longevity of bacterial viability [36]. The effects of anionic (SDS) and cationic (CTAB) surfactants on the stability of binary bacterial co-aggregates, as studied by [20], revealed that both SDS and CTAB promoted bacterial coaggregation at lower concentrations. However, complete deflocculation of the co-aggregates occurred at higher concentrations of 1 mg/ml SDS and 0.3 mg/l CTAB. Despite the SDS-CTAB pretreatment, the sludge maintained its hydrophobic character and displayed significant co-aggregation of bacteria, retaining 96% viability over a three-month period.

A group of potent microorganisms based on *in vitro* compatibility studies was assembled for comparison [20]. Nine strains were categorized under the genera *Bacillus*, *Streptomyces*, *Azotobacter*, and *Frauteria*. Previous studies [37, 38] reported comparable findings using *Actinomycetes*, fungi, and bacteria. Eight substrates of rice grain, sorghum grain, cotton cake, wheat straw, sawdust, sugarcane bagasse, and wheat bran were utilized by [39] to mass multiply bioagents, such as *Trichoderma harzianum* and *Gliocladium virens*. In sorghum grains, these bioagents showed good development after being transferred to other carriers. Previous researchers have observed similar outcomes using various carriers [40].

Another study assessed for plant growth-promoting rhizobacteria (PGPR) traits, such as indole-3-acetic acid (IAA) production, ammonia and hydrogen cyanide (HCN) production, and phosphate solubilization [25]. Furthermore, they were screened for *in vitro* salt (NaCl) tolerance and Na^+^ uptake patterns, and two stress-tolerant rhizobacteria, *Bacillus pumilus* and *Bacillus subtilis* showed all PGPR traits with tolerance to salinity.

The majority of PGPR *Actinomycetes* synthesize IAA, which is responsible for the increased number of adventitious roots that help plants take up a large volume of nutrients and absorb water, whereas increased root exudates in turn benefit the bacteria. The maximum phosphate solubilization activity was detected in *Streptomyces* sp. WA-1 (72.13 mg/100 ml). Moreover, previous research has reported 83.3, 58.9, and 39 mg/100 ml phosphate solubilization by *Streptomyces cavourensis*, *Streptomyces griseus*, and *Micromonospora aurantiaca*, respectively [41].

The best strains from each genus, *Azospirillum lipoferum* VAZS-18, *Azotobacter chroococcum* VZB-6, *Bacillus megaterium* VBA-2, and *Pseudomonas fluorescens* VPS-19, were used to create a carrier-based biofertilizer consortium, which was tested for suitability as a healthy inoculation practice for medicinal crops. In addition to producing effective strains of inoculants, maintaining the viability of microbial cells at a satisfactory level for a longer storage period is equally important [19]. The viable cell counts of all carrier materials examined declined over the course of five weeks at room temperature [42]. At approximately 28°C, coriander husk material produced the greatest results with the highest viable cell count. The ideal storage temperature for these PGPRs was 28°C.

The number of surviving individuals in various carrier materials was lower at 25°C than at 40°C. For *Azospirillum*, *Azotobacter*, *Bacillus*, and *Pseudomonas*, PGPR strains were estimated using single inoculant and consortium preparations for *Azospirillum*, *Azotobacter*, *Bacillus*, and *Pseudomonas*. At 40°C, the PGPR population in the pressmud, vermiculite, and alginate beads created with a single inoculant significantly decreased, averaged at 35°C, and peaked at 25°C [19, 43]. During a 6-month storage period, *Aspergillus flavus* NRRL 30793 propagules decreased more at 25°C than at 4°C [44]. In line with this, our findings demonstrated that while room temperature was appropriate for brief storage times, *Actinomycetes*-isolated strains could withstand 35°C during shelf-life studies for individual strains in long-term storage, apart from room temperature.

The findings of [11], who reported that peat, wheat bran, rice husks, vermiculite, clay, and alginate can be utilized as effective carriers, are consistent with our findings. It is important to note that sterilized carriers typically have substantially longer shelf life and can support bigger populations. Additionally, they demonstrated that, in comparison with autoclaving at 121°C, gamma irradiation almost completely preserved the chemical and physical properties of the material, making it the most appropriate method of carrier sterilization [45].

By studying [46], we will be able to explain the findings of software and hardware design, as well as the outcomes of device performance testing on every sensor that will be presented on the Blynk monitoring application. In addition, the application of each sensor was tested. We will review the features of the widget and its functionality in the Blynk application before testing starts. Blynk application design for biofertilizer storage can be found in [47]. Fakharulrazi and Yakub monitored the composting of food waste using a sensor-based application using a Blynk app for external parameter sensing [48].

## Conclusion

In conclusion, C_1_ showed the best overall survival under both temperature conditions, whereas C_2_ displayed the steepest decline. C_3_ showed a moderate reduction in the CFU counts. The packed C_1_ consortia formulation, using a dynamic NPK-rich carrier material, exhibited exceptional performance in maintaining strain viability at 28±2°C and 35±2°C temperature conditions. The accuracy of biotic and abiotic factors in the C_1_ consortia (*Streptomyces rameus* KAC3, *Streptomyces calvus* KAC10, and *Streptomyces werraensis* KAC11) was assessed through manual strain population counts and physical factor analysis using IoT devices. Over six months, the physical factors inside the packed product, such as temperature, humidity, CO_2_, and moisture, were monitored digitally and recorded against varying external environmental conditions, maintaining 100% viability of the C_1_ consortium strains by verifying the viable count for individual strains in different carrier materials. A consortium-based combined-carrier material formulation was studied for a 6-month duration. The C_1_ consortium-based formulation was highly efficient and stable at different temperatures, highlighting its potential as an eco-friendly bioinoculant for sustainable agriculture.

## Figures and Tables

**Table 1:** Properties of locally available cheapest carrier material.

S. No	Carrier materials	Collected area	Location	Element presents	Uses
1.	Wood ash [9]	Gopalapuram brick industry	Lat - 9.38955o N and Long - 77.63588o E	Macro elements (Ca, K, P, and S) Microelements (Zn, Cu, and Mn)	Fertilizers for soil and plants.
2.	Charcoal [16]	Waste management unit, KARE campus, Krishnankoil- 626126	Lat - 9.5747° N and Long - 77.6798° E	Elemental carbon	Improves growth. Quick absorption and water-holding capacity is high. Retains soil moisture.
3.	Eggshell powder [17]			95% Calcium carbonate and various macro and micro-nutrients (Mg, K, Fe and P)	Promotes plant growth. Stimulates root development.
4.	Vermi-cost [18, 19]	Crop cafeteria, Department of Agriculture, KARE, Krishnankoil.		Macro elements (Ca, N, K, P, Mg and S) Microelements (Zn, Cu, and Mn)	Improves soil texture, aeration, and increases water retention capacity.
5.	Adhesive material (surfactant) - SDS and *CTAB* [20]	Department of Chemistry, KARE, Krishnankoil.		SDS (anionic surfactant) and CTAB (cationic surfactant)	Used as adhesive particles between microorganism and carrier materials.

**Table 2:** Genbank details of collected PGP Actinomycetes.

Isolation source	Area	GPS location	Collected Actinomycetes	Isolates	NCBI Accession number
Dumping yard	Srivilliputtur Municipal Waste dumps area, Thilakulam.	Lat- 9.500981° & Long- 77.654466°	*Streptomyces rameus*	KAC3	PP550146
			*Streptomyces bangladeshensis*	KAC4	PP177363
			*Streptomyces mutabilis*	KAC6	PP177364
Alluvial soil	Atthi Kovil	Lat- 9.61263° & Long- 77.545506°	*Microbacterium arabinogalactanolyticum*	KAC8	PP177365
			*Streptomyces calvus*	KAC10	PP177366
Red Soil	KARE Campus, Krishnankoil.	Lat- 9.57287° & Long- 77.6780276°	*Streptomyces werraensis*	KAC11	PP177374
			*Streptomyces gancidicus*	KAC12	PP177389

KARE – Kalasalingam Academy of Research and Education, Krishnankoil; NCBI - National Center for Biotechnology Information

**Table 3 T3:** Composition of the Actinomycete isolation agar (AIA) medium.

Chemical Name	Composition (g/l)
Sodium caseinate	2.0
L-Asparagine	0.1
Sodium propionate	4.0
Dipotassium phosphate	0.5
Magnesium sulphate	0.1
Ferrous sulphate	0.001
Agar	15
Final pH (at 25°C) 8.1±0.2	

**Table 4:** Sensor uses and applications.

S. No	Sensor	Uses and their applications
1.	S410 pH Sensor [30]	Acidity and alkalinity of water quality monitoring in agriculture and industry purpose.
2.	DS18B20 Temperature Sensor [31]	Sensor provides accurate temperature readings for environmental monitoring.
3.	DOL119 CO_2_ Sensor [32]	Monitors CO_2_ levels to assess indoor air quality and ventilation.
4.	SHT20 Humidity Sensor [33]	Tracks relative humidity for climate control and mold prevention to maintain comfort levels in indoor environments.
5.	SEN0308 Moisture Sensor [34]	Detects soil moisture for optimal agricultural irrigation and plant health by optimizing water usage.

**Table 5:** Physical and chemical properties of carrier materials.

Carrier material		pH	EC (dSm^-1^)	Available N (%)	Available P (%)	Available K (%)	Total carbon (%)	C:N
Wood ash		11.1	13.9	1.72	0.86	3.6	18.0	10.5
Charcoal		8.5	19.5	0.93	23.68	0.80	67.18	6.5
Eggshell powder		6.5	0.1	-	0.4	0.3	-	-
Vermi-cost		7.7	6.88	3.5	0.71	0.6	18.5	5.51
Surfactant	SDS	6.5	-	-	-	-	-	-
	CTAB	6.0	-	-	-	-	-	-

**Table 6:** Qualitative assessments of PGP traits for Actinomycete isolates.

	KAC3	KAC4	KAC6	KAC8	KAC10	KAC11	KAC12
Potassium solubilization	++	+	-	+	++	+	+
Phosphate Solubilization	+++	++	+	-	+++	++	++
Siderophore Production	+++	-	+++	++	+++	+++	++
IAA Production	+++	++	+	++	++	+++	++

(-) No Growth; (+) Normal Growth; (++) Light zone appearance; (+++) Clear zone appearance.

**Table 7:** Consortium selections of Actinomycete isolates from compatibility assessment.

S. No	Combined culture	Consortium	Consortium
1.	KAC3 - KAC10 - KAC11	Compatible	C_1_
2.	KAC3 - KAC8	Non-Compatible	
3.	KAC4 - KAC8 - KAC10	Compatible	C_2_
4.	KAC8 - KAC11	Compatible	
5.	KAC6 - KAC10	Compatible	
6.	KAC6 - KAC8	Compatible	
7.	KAC11 - KAC12	Compatible	C_3_

**Table 8:** Individual Actinomycete isolates in combined carrier material (CFU/g) at 28±2°C up to 6 months.

Month	KAC3	KAC4	KAC6	KAC8	KAC10	KAC11	KAC12
0	10.17±0.07^g^	10.23±0.07^g^	10.07±0.03^g^	10.07±0.03^g^	10.10±0.06^g^	10.20±0.12^g^	10.17±0.01^g^
1	9.96±0.01^f^	9.02±0.01^f^	9.02±0.01^f^	9.43±0.02^f^	9.91±0.01^f^	9.56±0.03^f^	9.09±0.05^f^
2	9.79±0.01^e^	8.45±0.01^e^	8.68±0.02^e^	9.10±0.02^e^	9.64±0.02^e^	9.27±0.02^e^	8.74±0.03^e^
3	9.64±0.01^d^	8.10±0.02^d^	8.26±0.02^d^	8.79±0.02^d^	9.05±0.02^d^	8.94±0.01^d^	8.44±0.02^d^
4	9.05±0.01^c^	7.68±0.02^c^	7.89±0.02^c^	8.38±0.04^c^	8.74±0.02^c^	8.66±0.01^c^	8.10±0.02^c^
5	8.74±0.03^b^	7.39±0.04^b^	7.36±0.02^b^	7.90±0.01^b^	8.31±0.02^b^	8.39±0.02^b^	7.69±0.01^b^
6	8.53±0.04^a^	7.09±0.05^a^	7.02±0.02^a^	7.32±0.02^a^	8.04±0.03^a^	8.01±0.02^a^	7.36±0.02^a^

Data in the table represent the mean of consortium population from three replicates. The±symbol represents the values for standard error of means. Different letters represent the significant difference between treatments, using Duncan’s multiple range test (*p*=0.05).

**Table 9:** Individual Actinomycete isolates in combined carrier material (CFU/g) at 35±2°C up to 6 months.

Month	KAC3	KAC4	KAC6	KAC8	KAC10	KAC11	KAC12
0	10±0.06^g^	10.07±0.09^g^	9.83±0.09^g^	10.07±0.03^g^	10.13±0.03^g^	10±0.1^g^	10.07±0.07^g^
1	9.66±0.01^f^	9.19±0.01^f^	9.39±0.04^f^	9.22±0.02^f^	9.54±0.01^f^	9.56±0.01^f^	9.31±0.02^f^
2	9.37±0.01^e^	8.68±0.02^e^	9.02±0.03^e^	8.65±0.01^e^	9.24±0.01^e^	9.22±0.02^e^	9±0.02^e^
3	8.84±0.01^d^	8.23±0.02^d^	8.58±0.02^d^	8.22±0.02^d^	8.74±0.03^d^	8.69±0.01^d^	8.7±0.03^d^
4	8.23±0.02^c^	7.66±0.02^c^	8.03±0.02^c^	7.85±0.01^c^	8.29±0.02^c^	8.27±0.02^c^	8.32±0.02^c^
5	8.07±0.01^b^	7.29±0.02^b^	7.36±0.03^b^	7.32±0.01^b^	7.93±0.02^b^	7.83±0.03^b^	7.72±0.02^b^
6	7.83±0.02^a^	7.02±0.02^a^	6.46±0.04^a^	7.05±0.03^a^	7.64±0.02^a^	7.53±0.02^a^	7.13±0.02^a^

Data in the table represent the mean of consortium population from three replicates. The±symbol represents the values for standard error of means. Different letters represent the significant difference between treatments, using Duncan’s multiple range test (*p* = 0.05).

**Table 10:** Actinomycete consortia populations (CFU/g) in combined unique bioformulation.

Actinomycete consortia in combined carrier material at 28±2°C up to 6 months							
	0	1	2	3	4	5	6
C_1_	10.27±0.03^g^	9.97±0.01^f^	9.63±0.02^e^	9.32±0.02^d^	8.97±0.01^c^	8.74±0.02^b^	8.48±0.02^a^
C_2_	10.03±0.07^g^	9.65±0.02^f^	9.21±0.02^e^	8.78±0.02^d^	8.31±0.02^c^	7.82±0.02^b^	7.22±0.02^a^
C_3_	10.13±0.03^g^	9.79±0.01^f^	9.35±0.02^e^	9.04±0.02^d^	8.63±0.01^c^	8.11±0.01^b^	7.78±0.02^a^
Actinomycete consortia in combined carrier material at 35±2°C up to 6 months							
	0	1	2	3	4	5	6
C_1_	10.07±0.03^g^	9.83±0.02^f^	9.32±0.02^e^	8.8±0.02^d^	8.41±0.02^c^	8.03±0.02^b^	7.65±0.02^a^
C_2_	9.83±0.09^g^	9.38±0.01^f^	8.71±0.01^e^	8.11±0.01^d^	7.43±0.01^c^	7.03±0.03^b^	6.47±0.03^a^
C_3_	10.1±0.06^g^	9.62±0.02^f^	9.21±0.03^e^	8.62±0.05^d^	8.01±0.01^c^	7.42±0.02^b^	7.02±0.02^a^

Data in the table represent the mean of consortium population from three replicates. The±symbol represents the values for standard error of means. Different letters represent the significant difference between treatments, using Duncan’s multiple range test (*p* = 0.05).

## References

[1] Omar AF, Abdelmageed AH, Al-Turki A, Abdelhameid NM, Sayyed RZ, Rehan M (2022). Exploring the plant growth-promotion of four *Streptomyces* strains from rhizosphere soil to enhance cucumber growth and yield. Plants.

[2] Alexander M (1977). Introduction to Soil Microbiology.

[3] Hati KM, Swarup A, Dwivedi AK, Misra AK, Bandyopadhyay KK (2007). Changes in soil physical properties and organic carbon status at the topsoil horizon of a vertisol of central India after 28 years of continuous cropping, fertilization, and manure. Agric. Ecosyst. Environ..

[4] Kannan R, Ajay Kallapiran K (2022). A review on the agricultural waste residues management by different microbes. J. Agric. Ecol..

[5] Patil SA, Navale AM, Deokar CD, Patil DA (2021). Development and assessment of a microbial consortium for composting of organic waste. J. Pharmacogn. Phytochem..

[6] Franco-Correa M, Quintana A, Duque C, Suarez C, Rodrıguez MX, Barea JM (2010). Evaluation of *Actinomycete* strains for key traits related to plant growth promotion and mycorrhiza helping activities. Appl. Soil Ecol..

[7] FAO (1993). Technical Handbook on Symbiotic Nitrogen Fixation in Food and Agriculture 179.

[8] Maheshwari DK, Dubey RC, Agarwal M, Dheeman S, Aeron A, Bajpai VK (2015). Carrier-based formulations of biocoenotic consortia of disease-suppressive *Pseudomonas aeruginosa* KRP1 and *Bacillus licheniformis* KRB1. Ecol. Eng..

[9] Arora NK, Tiwari S, Singh R (2014). Comparative study of different carriers inoculated with nodule-forming and free-living plant growth-promoting bacteria suitable for sustainable agriculture. J. Plant Pathol. Microbiol..

[10] Duquenne P, Chenu C, Richard G, Catroux G (1999). Effect of carbon source supply and its location on competition between inoculated and established bacterial strains in sterile soil microcosm. FEMS Microbiol. Ecol..

[11] Abd El-Fattah DA, Eweda WE, Zayed MS, Hassanein MK (2013). Effect of carrier materials, sterilization method, and storage temperature on survival and biological activities of *Azotobacter chroococcum* inoculants. Ann. Agric. Sci..

[12] Somasegaran P, Hoben HJ Handbook for Rhizobia: Methods in Legume Rhizobium Technology.

[13] Stephens JH, Rask HM (2000). Inoculant production and formulation. Field Crops Res..

[14] Ferreira EM, Castro IV (2005). Residues of the cork industry as carriers for the production of legume inoculants. Silva Lusit..

[15] Wang HY, Shen LIU, Zhai LM, Zhang JZ, Ren TZ, Fan BQ (2015). Preparation and utilization of phosphate biofertilizers using agricultural waste. J. Integr. Agric..

[16] Gupta A, Bano A, Rai S, Sharma S, Pathak N (2022). Selection of carrier materials to formulate bioinoculant packages for promoting seed germination. Lett. Appl. NanoBioSci..

[17] Kannan R, Kallapiran KA (2024). Effect of eggshell powder on the growth performance of Okra (*Abelmoschus esculentus*) plant. Vegetos..

[18] Sekar KR, Karmegam N (2010). Earthworm casts as an alternative carrier material for biofertilizers: assessment of endurance and viability of *Azotobacter chroococcum*, *Bacillus megaterium* and *Rhizobium leguminosarum*. Sci. Hortic..

[19] Sangeetha D, Stella D (2012). Survival of plant growth-promoting bacterial inoculants in different carrier materials. Int. J. Pharm. Biol. Arch..

[20] Malik A, Kimchhayarasy P, Kakii K (2005). Effect of surfactants on the stability of *Acinetobacter johnsonii* S35 and *Oligotropha carboxidovorans* S23 co-aggregates. FEMS Microbiol. Ecol..

[21] Kannan R, Damodhran T, Pandey BK, Umamaheswari S, Rai R, Jha SK (2014). Isolation and characterization of endophytic plant growth-promoting bacteria (PGPB) associated with the sodicity-tolerant polyembryonic mango (*Mangifera indica* L.) root stock and growth vigor in rice under a saline sodic environment. Afr. J. Microbiol. Res..

[22] Walkley A, Black IA (1934). Examination of the Degtjareff method for determining soil organic matter and the proposed modification of the chromic acid titration method. Soil Sci..

[23] Pansu M, Gautheyrou J (2006). Handbook of soil analysis: mineralogical, organic and inorganic methods.

[24] Hussain F, Malik KA (1985). Evaluation of alkaline permanganate method and its modification as an index of soil nitrogen availability. Plant Soil.

[25] Damodaran T, Rai RB, Jha SK, Kannan R, Pandey BK, Sah V (2014). Rhizosphere and endophytic bacteria for induction of salt tolerance in gladiolus grown in sodic soils. J. Plant Interact..

[26] Chukwuneme CF, Babalola OO, Kutu FR, Ojuederie OB (2020). Characterization of *Actinomycete* isolates for plant growth promoting traits and their effects on drought tolerance in maize. J. Plant Interact..

[27] Al-Hussini HS, Al-Rawahi AY, Al-Marhoon AA, Al-Abri SA, Al-Mahmooli IH, Al-Sadi AM (2019). Biological control of damping-off of tomato caused by *Pythium aphanidermatum* by using native antagonistic rhizobacteria isolated from Omani soil. J. Plant Pathol..

[28] Singh J, Singh A, Upadhayay V, Khan A (2020). Comparative evaluation of developed carrier-based bioformulations bearing multifarious PGP properties and their effect on shelf life under different storage conditions.

[29] Zaveri KA, Amin MH, Amin MS, Patel MR (2021). IoT-based real-time low-cost home quarantine patient aid system using the Blynk app. In J. Phys. Conference Series, IOP Publishing.

[30] Kumar AA, Kumar SKN (2019). A comprehensive Review of pH- and nutrient-sensitive materials and methods for agricultural applications. Sensor Lett..

[31] Bachuwar VD, Shligram AD, Deshmukh LP (2018). Monitoring soil parameters using IoT and Android applications for smart agriculture. AIP Conference Proceedings,.

[32] Wang J, Niu X, Zheng L, Zheng C, Wang Y (2016). Wireless mid-infrared spectroscopy sensor network for automatic carbon dioxide fertilization in a greenhouse environment. Sensors.

[33] Liu J, Zhao Z, Jin J, Fang Z, Du L (2020). High-precision humidity sensor response time measurement based on the switchable chamber with the hydrophobic interface. IEEE Sensors J..

[34] Pereira GP, Chaari MZ, Daroge F (2023). IoT-enabled smart drip irrigation system using ESP32. IoT.

[35] Kaljeet S, Keyeo F, Amir HG (2011). Influence of carrier materials and storage temperature on the survivability of rhizobial inoculants. Asian J. Plant Sci..

[36] Ali MN, Chakraborty S, Paramanik A (2012). Enhancing the shelf life of kunapajala and shasyagavya and their effects on crop yield. Int. J. Bio-Resour. Stress Manag..

[37] Mishra DS, Kumar A, Prajapati CR, Singh AK, Sharma S (2013). Identification of compatible bacterial and fungal isolates and their effectiveness against plant disease. J. Environ. Biol..

[38] Gupta R, Bisaria VS (2013). Sharma, S. Bioinoculants: more than just plant growth-promoting agents. J. Endocyto Biol. Cell Res..

[39] Dawar S, Gaffar A (2003). Screening of substrates for mass production of biocontrol agents. Pakistan J. Bot..

[40] Gaur RB, Sharma RN, Sharma RR (2005). Shelf life of talc-based formulation of *Trichoderma* and soil application for biological control of dry root rot in chickpeas. J. Mycol. Pl. Pathol..

[41] Anwar S, Ali B, Sajid I (2016). Screening of rhizospheric Actinomycetes for various *in vitro* and *in vivo* plant growth promoting (PGP) traits and for agroactive compounds. Front. Microbiol..

[42] Arora S, Mukherji I, Kumar A, Tanwar RK (2014). Pesticide residue analysis of soil, water, and grain of IPM basmati rice. Environ. Monit. Assess..

[43] Bajpai PD, Gupta BR, Ram B (1978). Survival of *Rhizobium leguminosarum*in two carriers as affected by moisture and temperature conditions. Ind. J. Agrl. Res..

[44] Accinelli C, Ludovica SM, Abbas HK, Zablotowicz RM, Wilkinson JR (2009). Use of a granular bioplastic formulation for carrying conidia of a non-aflatoxigenic strain of *Aspergillus flavus*. Bioresour. Technol..

[45] Alok Kalra, Mahesh Chandra, Ashutosh Awasthi, Anil K Singh, Suman Preet S. Khanuja (2010). Natural compounds enhancing growth and survival of rhizobial inoculants in vermicompost-based formulations. Biol. Fertil. Soils.

[46] Isyanto H, Jumail J, Rahayu R, Firmansyah N (2021). Design of monitoring device for the process of organic waste decomposition into compost fertilizer and plant growth through smartphones based on Internet of Things Smart Farming. J. Electr. Technol. UMY.

[47] Serikul P, Nakpong N, Nakjuatong N (2019). Smart farm monitoring via the Blynk IoT platform: Case study: Humidity monitoring and data recording. Int. Conf. ICT Knowl. Eng..

[48] Fakharulrazi AN, Yakub F (2020). Designing an automated oster for food waste management with the implementation of Internet of Things. J. Sustain. Nat. Resour..

